# Antimicrobial Susceptibility of *Clostridioides difficile* in Spain: Multicenter Retrospective Cohort Study

**DOI:** 10.3390/antibiotics15020145

**Published:** 2026-02-02

**Authors:** María-Paz Ventero, María-Dolores Valverde-Fredet, Esperanza Merino, Rocío Herrero, Iryna Tyschkovska Germak, Miguel Rodríguez-Fernández, Jose-Manuel Ramos-Rincón, Maria Garcia, Elisabet Delgado-Sánchez, Miguel Nicolás Navarrete-Lorite, Concepcion Gil, María Tasias, Juan Jose Caston, David Vinuesa-Garcia, Cristina Gomez-Ayerbe, Francisco J. Martínez Marcos, Nicolas Merchante, Juan Carlos Rodríguez

**Affiliations:** 1 Microbiology Service, Alicante General University Hospital, Alicante Institute of Health and Biomedical Research (ISABIAL), 03010 Alicante, Spain; maripazvm@gmail.com (M.-P.V.); germak_iry@isabial.es (I.T.G.); rodriguez_juadia@gva.es (J.C.R.); 2 Infectious Diseases and Microbiology Clinical Unit, Valme University Hospital, Institute of Biomedicine of Seville (IBiS), University of Seville, 41004 Sevilla, Spain; fredet95@gmail.com (M.-D.V.-F.); herrero.delrio@gmail.com (R.H.); migrodfer92@gmail.com (M.R.-F.); nicolasmerchante@gmail.com (N.M.); 3 Clinical Medicine Department, Miguel Hernández University, 03202 Elche, Spain; merinoluc@gmail.com; 4 Infectious Diseases Unit, Alicante General University Hospital, Alicante Institute of Health and Biomedical Research (ISABIAL), 03010 Alicante, Spain; 5 Infectious Diseases Unit, Vega Baja University Hospital, 03314 Orihuela, Spain; garcia_marialop@gva.es; 6 Foundation for the Promotion of Health and Biomedical Research of the Valencian Community (FISABIO), 46020 Valencia, Spain; 7 Infectious Diseases Unit, Sant Joan D’Alacant University Hospital, 03550 Sant Joan D’Alacant, Spain; delgado_eli@gva.es; 8 Infectious Diseases Unit, Virgen Macarena University Hospital, 41009 Sevilla, Spain; miguelnnl1987@gmail.com; 9 Infectious Diseases Unit, Marina Baixa Hospital, 03570 Villajoyosa, Spain; cgilanguita@gmail.com; 10 Infectious Diseases Service, La Fe University and Polytechnic Hospital, 46026 Valencia, Spain; marionatasias@hotmail.com; 11 Infectious Diseases Unit, Reina Sofía University Hospital, 14004 Córdoba, Spain; juanj.caston.sspa@juntadeandalucia.es; 12 CIBER de Enfermedades Infecciosas (CIBERINFEC), Instituto Salud Carlos III (ISCIII), 28029 Madrid, Spain; 13 Infectious Diseases Unit, San Cecilio University Hospital, 18007 Granada, Spain; david.vinuesa.sspa@juntadeandalucia.es; 14 Infectious Diseases Unit, Virgen de la Victoria University Hospital, 29010 Malaga, Spain; cristina.gomez.ayerbe.sspa@juntadeandalucia.es; 15 Infectious Diseases Unit, Juan Ramón Jimenez University Hospital, 21005 Huelva, Spain; francisco.martinez@denf.uhu.es

**Keywords:** *Clostridioides difficile* infection, vancomycin, eravacycline, metronidazole, fidaxomicin, tigecycline, resistant isolates

## Abstract

**Background/Objetives**: The objective of this study was to determine the in vitro susceptibility profiles of clinical *Clostridioides difficile* isolates to metronidazole (MTZ), vancomycin (VAN), fidaxomicin (FDX), tigecycline (TGC), and eravacycline (ERV) in a multicenter Spanish cohort, and to evaluate their association with clinical factors. **Methods**: Strains were obtained from prospectively included patients in the ICD-ANCRAID-SEICV cohort (ClinicalTrials.gov ID: NCT04801862) in Andalucía and the Valencian Community between 1 January 2020 and 30 April 2023. Antimicrobial susceptibility testing was performed using E-test for MTZ, VAN, TGC, and ERV, and agar dilution for FDX. **Results**: The results were interpreted following EUCAST clinical breakpoints and ECOFF criteria. A total of 107 patients were included (median age 70 years; 65.4% women). Nearly half of the cases were community-acquired, 30% nosocomial, and the remainder healthcare-associated. Most infections were non-severe, and 32.7% experienced recurrence. Overall resistance levels were low: VAN and TGC each showed resistance in 2.8% of isolates, followed by MTZ (1.9%). Only one isolate was resistant to FDX (0.9%), and none to ERV. MIC90 values were low for all agents. Some resistant isolates displayed co-resistance and were recovered from patients with prior antibiotic exposure. Among the seven patients carrying resistant strains, most were women, and the cases were predominantly community-acquired. Clinical characteristics, including age, comorbidity, infection origin, and severity, did not differ from those with susceptible isolates. All patients achieved clinical cure without recurrent infection. No association was found between elevated MIC values and recurrence or greater severity. **Conclusions:** FDX and ERV demonstrated excellent in vitro activity. Resistance to MTZ, VAN, and TGC was uncommon but detectable. Findings highlight the need for continued antimicrobial resistance surveillance and evaluation of its potential clinical impact.

## 1. Introduction

*Clostridioides difficile* is a clinically significant pathogen responsible for severe gastrointestinal infections, which may lead to life-threatening conditions. This infection imposes a substantial burden on healthcare systems, as well as notable social and economic impacts [1]. Currently, international guidelines recommend fidaxomicin (FDX) and vancomycin (VAN) as first-line treatments [2,3,4]. Due to its higher recurrence rates, metronidazole (MTZ) is no longer considered a first-line therapy, but it may be used in situations where first-line options are unavailable or contraindicated [2,3]. In Europe, there is significant variation in management strategies: VAN is still the most common first-line agent, followed by FDX. Although MTZ is currently recommended for selected low-risk cases, it continues to be widely used in clinical practice [5].

Reduced susceptibility or resistance to all three antibiotics has been reported in *C. difficile* isolates [6], with a resistance rate of 1.0% for VAN and MTZ and 0.08% for FDX [7]. However, resistance rates vary widely across studies, particularly for MTZ and VAN, largely depending on the geographic region. One pan-European study showed resistance rates of 2.18% for VAN and 3.16% for MTZ, with no isolates resistant to FDX detected [8]. In contrast, an Australian study reported VAN resistance rates of 5.7% and no isolates resistant to MTZ or FDX [9]. Observed resistance rates also vary by the laboratory methods employed (e.g., agar dilution, E-test, broth microdilution…) [10]. Such heterogeneity in study design and testing procedures complicates comparisons and may partly explain the inconsistent resistance rates reported in the literature.

Tigecycline (TGC) is an antibiotic with activity against *C. difficile*, used for treating severe *C. difficile* infections (CDI) [11]. Eravacycline (ERV) is a novel synthetic fluorocycline antibacterial agent approved for complicated intra-abdominal infections, showing in vitro activity against a wide variety of *C. difficile* strains [12]. Different authors have reported clinical strains of *C. difficile* with varying susceptibility profiles to MTZ, VAN, FDX, and TGC, detecting resistance in a subset of strains, while newer antimicrobials, such as ERV, have demonstrated high in vitro activity and low or non-existent resistance rates for *C. difficile* [12,13].

The primary objectives of this study were to characterize the in vitro antimicrobial susceptibility profiles of *C. difficile* isolates to MTZ, VAN, FDX, TGC, and ERV and to identify specific resistance patterns. Secondary objectives were to evaluate risk factors associated with antimicrobial resistance, describe minimum inhibitory concentration (MIC) variations in patients with recurrent CDI, and assess clinical outcomes according to MIC values.

## 2. Results

### 2.1. Participant Characteristics

The entire cohort included 413 patients during the study period. Faecal samples were collected from 292 (70.7%) patients. A random selection of 50% of these samples was used to perform the antibiotic susceptibility testing, from which a total of 107 (73.3%) viable strains of *Clostridium difficile* were obtained. Participants were mainly women (65.4%, 70/107); they had a median age of 70 years (IQR 60–81) and a median Charlson Comorbidity Index of 3. More than half (61.4%, 55/107) had been hospitalized within the previous three months, and 9.3% (10/107) had undergone surgery.

Nearly half the CDI episodes were community-acquired (47.7%, 51/107), 29.9% were nosocomial, and 22.4% (24/107) were healthcare-associated, and only two were from patients with previous CDI episodes (1.9%, 2/107). Most cases were classified as mild or moderate (74.7%, 80/107), while 23.2% (25/107) were severe and 1.9% (2/107) were fulminant. The binary toxin was detected in 65.4% (70/104) of isolates.

Prior antibiotic exposure was almost universal (93.5%, 100/107), with a median of two treatment courses, and 65.4% (70/107) of patients had received proton pump inhibitors before CDI onset. During the CDI episode, VAN was the most prescribed agent (84.1%, 90/107), followed by FDX (21.5%, 23/107) and MTZ (5.6%, 6/107). Bezlotozumab was administered in 10.3% (11/107) of cases, and the median hospital stay was 16 days (IQR 9–18). The recurrence rate was 19.6% (81/413) in the entire cohort and 32.7% (35/107) among the patients included in this study. Table 1 presents the clinical features of the study participants.

### 2.2. Antimicrobial Susceptibility Testing and Characteristics of Patients with Resistant Isolates

A total of 107 *C. difficile* isolates were evaluated for their susceptibility to MTZ, VAN, FDX, TGC, and ERV. Table 2 summarizes MIC values, including MIC50 and MIC90, along with resistance rates.

Overall, all antibiotics demonstrated high in vitro activity against *C. difficile*. Resistance was rare, observed in only one isolate (0.9%, 1/107) for MTZ and FDX, and in three isolates (2.8%, 3/107) for VAN, and the same percentage for TGC-resistant isolates. No resistant isolates were detected to ERV. FDX and TGC exhibited similarly low MIC90 values. ERV showed the greatest potency, with an MIC50 of 0.006 mg/L and a MIC90 of 0.0128 mg/L. Of the seven resistant isolates, one showed resistance to both MTZ and VAN, two only to VAN, one only to MTZ, one to FDX and TGC, and two only to TGC (Table 3).

Five of the seven cases (71.4%, 5/7) occurred in women and originated in community settings. None had experienced a previous episode of CDI. All but one (85.7%, 6/7) had underlying medical conditions, most frequently active neoplasia, chronic steroid therapy, or metabolic disease. No recurrences were observed, and all episodes achieved clinical cure (Table 4).

### 2.3. Comparison Between Resistant Versus Susceptible Isolates and Association Between MIC Values, Recurrence, and Disease Severity

Table 5 compares patients with antibiotic-resistant versus susceptible *C. difficile* isolates. No statistically significant differences were observed between the two groups for any of the variables analyzed, although those with resistant isolates tended to be younger (median age 58 vs. 70 years, *p* = 0.10) and had slightly lower comorbidity scores (Charlson index 2 vs. 3, *p* = 0.16). The distribution by sex, origin of infection (community-acquired, nosocomial, or healthcare-associated), and clinical severity was similar in both groups.

Likewise, previous exposure to antibiotics or proton pump inhibitors, hospitalization rates, and outcomes—including recurrence and length of hospital stay—did not differ significantly between groups.

Table 6 shows the relationship between antimicrobial MIC categories and clinical outcomes. No statistically significant associations were observed between MIC values for VAN, MTZ, FDX, TGC, or ERV and either CDI recurrence or disease severity. Similarly, antimicrobial resistance was not related to worse clinical outcomes.

## 3. Discussion

The resistance rates obtained in this study were very low, and the few resistant strains were not associated with episodes of recurrence or severity. Our cohort was made up of older adults who frequently had several comorbidities and a recent history of hospitalization, characteristics similar to those found in other epidemiological studies of CDI [14,15]. However, the recurrence rate in our sample (32.7%) was higher than that of the entire cohort (19.6%) and that reported elsewhere (around 20%) [16,17]. The reason may be that this study included only patients with stool samples from which a viable strain of *C. difficile* was obtained, suggesting that the recovery rate of viable strains is higher in patients with recurrent CDI episodes.

In terms of susceptibility patterns, VAN and TGC showed the highest resistance rates (2.8% each). MTZ and FDX exhibited low resistance rates (0.9% each), and no ERV-resistant isolates have been reported. In Spain, an observational study reported VNC resistance in 4.1% and MTZ resistance in 3.5% of clinical isolates, higher than the values observed in our study [18]. In contrast, a study conducted in Israel reported a VAN resistance rate of approximately 1.6%, which is closer to the figure we observed [19]. In the United States, the latest surveillance study published found low resistance rates for TGC and VNC (0.7% of resistant isolates) as well as for MTZ (0.3%), and a low MIC90 for FDX (0.5 µg/mL) [20]. In the pan-European ClosER study, surveillance data indicated that resistance to VAN and MTZ was under 4% and to TGC and FDX, even lower (<0.5%), although rates vary depending on the country and ribotype [14]. MIC values can also vary depending on the laboratory method used to calculate them [21], increasing the observed variations between different geographical locations and preventing robust between-study comparisons.

For ERV, our study shows extremely low MIC50/90 values (0.006/0.0128 mg/L) and no resistant isolates. In a cohort of 234 clinical isolates representing major epidemic and emerging ribotypes, including RT027, ERV demonstrated the lowest MICs among tested agents (MIC50/90 values, ≤0.0078/0.016 mg/L), again with no resistant isolates identified [12]. This potent activity was observed regardless of ribotype or the presence of tetM/tetW resistance genes, indicating that ERV’s efficacy is not compromised by common tetracycline resistance mechanisms [22]. Comparative studies have shown that ERV’s MICs are consistently lower than those of FDX, MTZ, and VAN, and its minimum bactericidal concentrations (MBCs) are also lower than VAN for all ribotypes tested [23]. Notably, ERV retains activity against multidrug-resistant anaerobic pathogens, including *C. difficile* strains with reduced susceptibility to standard therapies [23]. Global surveillance of Gram-positive pathogens further supports the stability of ERV MICs over time and across regions, with no substantive increase in resistance detected [24].

In our study, no host-related factors were identified as being associated with antimicrobial resistance in *C. difficile*, which is consistent with the recent literature. A multicenter study in the United States demonstrated that decreased antibiotic susceptibility in *C. difficile* is not directly associated with disease severity or recurrence, and host-related factors are not linked to antimicrobial resistance but rather to the presence of specific toxins and ribotypes [25]. In this line, adjunctive strategies targeting toxin-mediated mechanisms have emerged as important considerations in preventing CDI recurrence, particularly when antimicrobial susceptibility alone does not predict recurrence risk. For example, bezlotoxumab—a human monoclonal antibody against toxin B—has demonstrated effectiveness in reducing recurrence rates when administered during standard-of-care, antibiotic therapy for a primary CDI episode [26]. Furthermore, no significant differences in antimicrobial susceptibility have been reported between recurrent and non-recurrent episodes, or between relapses and reinfections [27]. In North America, a recent study suggests that reduced VAN susceptibility is associated with lower cure rates and sustained clinical response, although no direct relationship with recurrence has been demonstrated [28].

On the other hand, our results show no association between higher MIC values and recurrence or more severe forms of infection, in contrast with previous studies. Notably, several isolates in our cohort exhibited markedly elevated MICs to first-line therapies, including vancomycin and fidaxomicin, yet these findings were not associated with adverse clinical outcomes. Specifically, isolates 1 and 2 showed very high vancomycin MICs, while isolate 5 presented an increased fidaxomicin MIC; nevertheless, all corresponding patients achieved clinical cure with the administered therapy. This apparent discordance between in vitro susceptibility and clinical response has been previously described in *CDI* and may be explained by the exceptionally high intraluminal concentrations achieved by orally administered vancomycin and FDX, which often exceed reported MICs by several orders of magnitude. Moreover, current susceptibility breakpoints for *C. difficile* are largely based on epidemiological cut-off values rather than robust correlations with clinical outcomes, limiting their predictive value for treatment failure.

For example, Valsecchi et al. reported that although isolates from patients with recurrence showed increased MICs, only higher VAN MICs were associated with greater 28-day mortality, but not with recurrence itself [29]. Other studies have posited a possible link between decreased susceptibility to VAN and lower initial cure rates and sustained clinical response, though without a direct relationship to recurrence [30,31]. Similarly, previous research has found no correlation between antimicrobial susceptibility and recurrence, nor significant differences in MTZ or VAN susceptibility among relapse, reinfection, and single-episode cases [27,31,32].

Conversely, the literature emphasizes that CDI recurrence is more strongly associated with factors such as intestinal dysbiosis, persistence of spores, and prior antibiotic exposure—which promote colonization and the recurrence cycle—rather than with antimicrobial resistance per se [33,34]. Increased expression of sporulation and adhesion-related genes in isolates from patients with recurrence may contribute to persistence, but a direct relationship with antimicrobial resistance has not been demonstrated [35].

The results obtained in this study demonstrate that, although antimicrobial susceptibility testing for *C. difficile* is not routinely performed due to its technical complexity and limited clinical applicability, these tests are necessary to maintain continuous surveillance and monitor local and global resistance trends. Our data confirm that, from a health perspective, antibiotic sensitivity tests are of limited use, difficult to perform methodologically, and time-consuming for the laboratory. However, this type of testing is very important from a public health and epidemiological surveillance perspective, so it is necessary to continue conducting periodic monitoring to determine the rates of *C. difficile* resistance in our environment.

A major strength of our study is its multicenter design, which provides a representative overview of *C. difficile* antimicrobial susceptibility across different hospitals in Spain. In addition to assessing the three conventional therapeutic agents—VAN, FDX, and MTZ—we also evaluated TGC and the novel fluorocycline ERV, thereby broadening the antimicrobial spectrum assessed and contributing data on emerging therapeutic alternatives.

However, some limitations should be acknowledged. First, the sample size (107 isolates) and selection method (only 50% from randomized selection) may not be sufficient to detect low-prevalence patterns of resistance or regional variability. Furthermore, the inability to obtain a fecal sample from all the patients, combined with the failure to obtain a viable strain from all selected stool samples collected, introduces a risk of selection bias. In addition, no molecular analyses were performed to identify specific resistance genes or mechanisms. All isolates originated from Spanish centers, which may limit the generalizability of the findings to other geographic regions. Additionally, the use of different susceptibility testing methods (Etest vs. agar dilution) could influence MIC comparability. Finally, the cross-sectional design precludes evaluation of temporal trends in resistance.

Despite these limitations, this multicenter study provides valuable and up-to-date information on the antimicrobial susceptibility of *C. difficile* in our setting, where such analyses are rarely conducted in routine clinical practice.

## 4. Materials and Methods

### 4.1. Study Design and Population

This multicenter, retrospective, observational cohort study included patients aged 18 years or older from nine participating hospitals (Table 7) with a microbiologically confirmed diagnosis of CDI. The study period was from 1 January 2020 to 30 April 2023. Included patients were from the ICD-ANCRAID-SEICV cohort (ClinicalTrials.gov ID: NCT04801862) [36], a prospective, multicenter cohort study recruiting all consecutive adult patients with a new diagnosis of CDI. Consecutive patients from the ICD-ANCRAID-SEICV cohort (ClinicalTrials.gov ID: NCT04801862) who presented a viable *C. difficile* strain after stool sample culture were included.

### 4.2. Diagnosis and Definitions

Stool samples from patients with CDI diagnoses were processed in the microbiology laboratories of the participating hospitals (Table 7). An episode of CDI was defined as the presence of diarrhea and the detection of free toxins using enzyme immunoassay (EIA), or by nucleic acid amplification test (NAAT), in line with the criteria of the European Centre for Disease Prevention and Control (ECDC) [37] and the European Society of Clinical Microbiology and Infectious Diseases (ESMICD) [2,38].

The origin of CDI episodes was classified as (i) community-acquired or (ii) healthcare-associated, according to ECDC criteria [37], or as (iii) nosocomial, following Infectious Diseases Society of America (IDSA) guidance [39]. The patients were followed for 8 weeks. Severe CDI and recurrence were defined based on ESMICD and IDSA publications [38,39].

### 4.3. Clinical Data

Demographic and clinical data were collected from patients’ electronic medical records. Variables were age, sex, comorbidities, recurrence of CDI, and prior exposure to proton pump inhibitor or antibiotics. Additional variables included hospital admission in the 12 months before study inclusion, history of surgery, hospitalization, length of stay, and ICU admission during the CDI episode, severity of the episode, Binary toxin, treatment received for CDI, and classification of infection as community-acquired, nosocomial, or healthcare-associated CDI. Data were collected and managed using REDCap electronic data capture tools (REDCap 15.0.37), hosted at Vanderbilt University [40,41].

### 4.4. Isolation of C. difficile Strains

Fecal samples were cryopreserved at −80 °C. To isolate *C. difficile* strains, samples were thawed at 4 °C and plated on chromogenic medium (CHROMID *C. difficile*, bioMérieux, Marcy-l’Etoile, France) to select *C. difficile* colonies. Plates were incubated at 37 °C for 48 h under anaerobic conditions. Colonies suspected of being *C. difficile* were identified by MALDI-TOF (MALDI Biotyper, Bruker, Billerica, MA, USA). Colonies confirmed as *C. difficile* were subcultured on Schaedler agar, supplemented with 5% sheep blood (SCS, bioMérieux) to obtain pure isolates, which were then cryopreserved at −80 °C.

### 4.5. Susceptibility Testing for VAN, MTZ, TGC, and ERV

Cryopreserved strains were cultured on chromogenic medium (CHROMID *C. difficile*, bioMérieux) for identification control, and on Schaedler agar (SCS, bioMérieux) at 37 °C for 48 h under anaerobic conditions. Biomass obtained from Schaedler agar was used to prepare bacterial suspensions in sterile saline solution, which were then adjusted to a 1 McFarland standard. These suspensions were inoculated onto fastidious anaerobe agar plates to determine antibiotic susceptibility using the Epsilometer-test (E-test) technique with drug-specific commercial strips (bioMérieux). Plates were incubated at 37 °C for 48 h under anaerobic conditions, after which the minimum inhibitory concentrations (MICs) were determined.

### 4.6. Susceptibility Testing for FDX

E-test strips for FDX are not available, so an alternative method using agar dilution was employed. Agar plates were prepared with 20 mL of *C. difficile* selective agar base (Oxoid) and distributed into Petri dishes. After autoclaving, 5% defibrinated horse blood (Oxoid) and fixed concentrations of FDX were added prior to pouring the agar. Once solidified, 5 μL of a 1 McFarland *C. difficile* suspension was inoculated onto each plate and incubated anaerobically for 48 h. FDX was initially dissolved in 100% dimethyl sulfoxide (DMSO), and subsequent dilutions were made in sterile saline solution. Final concentrations tested ranged from 0.01 to 32 mg/L. MIC was defined as the lowest concentration that completely inhibited visible bacterial growth [42,43].

### 4.7. Analysis and Visualization

Two technicians independently read the plates, and any discrepancies were resolved by the senior microbiologist (JCR). EUCAST clinical breakpoints [21] were used to interpret susceptibility to VAN, FDX, and MTZ. For ERV and TGC, EUCAST epidemiological cutoff values (ECOFFs) were applied, as clinical breakpoints for these antibiotics are not yet available.

### 4.8. Statistical Analysis

Descriptive statistics were applied to summarize the characteristics of the isolates, including MIC50, MIC90, MIC range, number of resistant isolates, and percentage of resistance. Non-parametric continuous variables were expressed as median and interquartile range (IQR), and categorical variables as absolute frequencies and percentages. The prevalence of antimicrobial resistance was calculated by dividing the number of resistant isolates by the total number of isolates tested. Comparisons between patients with resistant versus susceptible isolates were performed using the Mann-Whitney U test for continuous variables and Fisher’s exact test for categorical variables.

Clinical outcomes were recurrence (yes/no) and severity of CDI (mild/moderate, severe, fulminant). Isolates were further stratified according to MIC values and categorized into tertiles for each antibiotic tested. Comparisons of clinical outcomes across MIC categories (2 × 3 contingency tables) were conducted using the Chi-square test. A two-tailed *p*-value of less than 0.05 was considered statistically significant. Statistical analyses were performed using IBM SPSS Statistics, version 24.0 (IBM Corp., Armonk, NY, USA).

### 4.9. Ethical Considerations

This study was designed and performed according to the Declaration of Helsinki and was approved by the Valme University Hospital Ethics Committee (Ref. 1254-N-20).

## 5. Conclusions

In this study, FDX and ERV exhibited strong in vitro activity against *C. difficile*, with very low MIC values and no evidence of resistance. Resistance to MTZ, VAN, and TGC was uncommon but relevant from an epidemiological standpoint. The presence of multidrug-resistant isolates, although rare, underscores the importance of ongoing susceptibility surveillance and antibiotic stewardship. Furthermore, the excellent in vitro performance of ERV highlights its potential role as an alternative agent for difficult-to-treat or multidrug-resistant *C. difficile* infections.

## Figures and Tables

**Table 1:** Characteristics of patients with *Clostridioides difficile* infection (CDI), N = 107.

Variable		Value
Demographics		
Age in years, median (IQR)		70 (60–81)
Sex, N(%)	Male	37 (34.6%)
	Females	70 (65.4%)
Clinical history and characteristics		
Charlson Comorbidity Index, median (IQR)		3 (2–4)
Previous hospitalization (<12 months), N (%)		55 (61.4%)
Previous surgery (<3 months), N (%)		10 (9.3%)
Previous episodes of CDI, N (%)		2 (1.9%)
Current episode of CDI, N (%)		
Community-acquired		51 (47.7%)
Nosocomial		32 (29.9%)
Healthcare-associated CDI		24 (22.4%)
Hospitalization		80 (74.8%)
Length of stay in days, median (IQR)		6 (9–18)
Binary toxin		70 (65.4%)
ICU admission		2 (1.9%)
Clinical outcomes		
Recurrence rate, N (%)		35 (32.7%)
	1 episode	29 (27.1%)
	2 episodes	6 (5.6%)
Fulminant CDI colitis		2 (1.9%)
Severe		25 (23.2%)
Mild/moderate		80 (74.7%)
Previous treatments (<3 months prior to inclusion in study), N (%)		
Proton pump inhibitors		70 (65.4%)
Antibiotics		100 (93.5%)
Number of cycles, median (IQR)		2 (1–2)
Previous use of vancomycin		2 (1.9%)
Previous use of metronidazole		10 (9.3%)
Previous use of fidaxomicin		0 (0.0%)
Previous use of tetracycline		4 (3.7%)
Therapies for the current CDI episode, N (%)		
Vancomycin,		90 (84.1%)
Fidaxomicin,		23 (21.5%)
Metronidazole,		6 (5.6%)
Bezlotoxumab		11 (10.3%)
Tigecycline,		1 (0.9%)

CDI: *Clostridioides difficile* infection; IQR: interquartile range.

**Table 2:** Antimicrobial susceptibility of *Clostridioides difficile* isolates.

Antimicrobial	MIC Range (mg/L)	MIC_50_ (mg/L)	MIC_90_ (mg/L)	N (%) of Resistant Isolates ^a^
Vancomycin	0.064–512	0.380	0.500	3 (2.8%)
Metronidazole	0.016–512	0.064	0.250	1 (0.9%)
Fidaxomicin	0.030–2	0.125	0.260	1 (0.9%)
Tigecycline	0.008–16	0.016	0.032	3 (2.8%)
Eravacycline	0.002–0.250	0.006	0.0128	0 (0.0%)

MIC: minimum inhibitory concentration ^a^ Resistance is defined as MIC > 2 mg/L for vancomycin, >2 mg/L for metronidazole, >0.5 mg/L for fidaxomicin, and non-wild type isolates defined as MIC > 0.25 mg/L for tigecycline (epidemiological cutoff value), >0.25 mg/L for eravacycline (epidemiological cutoff value).

**Table 3:** Minimum inhibitory concentrations (MIC) (mg/L) of resistant ^a^ *C. difficile* isolates to metronidazole (MTZ), vancomycin (VAN), fidaxomicin (FDX), tigecycline (TGC), and eravacycline (ERV).

	MTZ (MIC)	VAN (MIC)	FDX (MIC)	TGC (MIC)	ERV (MIC)
Isolate 1	R (>256)	R (>256)	S (0.25)	S (0.25)	S (0.25)
Isolate 2	S (0.125)	R (128)	S (0.06)	S (0.064)	S (0.006)
Isolate 3	S (0.125)	R (3)	S (0.03)	S (<0.016)	S (0.012)
Isolate 4	R (>256)	S (0.38)	S (0.06)	S (0.032)	S (0.006)
Isolate 5	S (0.047)	S (0.5)	R (2)	R (0.94)	S (0.001)
Isolate 6	S (0.094)	S (0.38)	S (0.012)	R (16)	S (0.125)
Isolate 7	S (0.125)	S (0.75)	S (0.03)	R (0.5)	S (0.032)

R: resistant, S: susceptible ^a^ Resistance is defined as MIC>2 mg/L for vancomycin, >2 mg/L for metronidazole, >0.5 mg/L for fidaxomicin, and non-wild type isolates defined as MIC > 0.25 mg/L for tigecycline (epidemiological cutoff value), >0.25 mg/L for eravacycline (epidemiological cutoff value).

**Table 4:** Characteristics of seven episodes with *C. difficile* antibiotic-resistant isolates.

Case	Age/Sex	Center (City)	Origin	Previous Episode of CDI	Underlying Condition	Antibiotics in Previous 3 Months (N Cycles)	Previous Treatment with PPI	Treatment	Recurrence ^a^	Clinical Cure ^b^
1	70/F	SCUH (Granada)	Community	No	Active neoplasm	CEP (N = 2) FQ (N = 1)	Yes	VAN	No	Yes
2	27/F	VVUH (Seville)	Healthcare associated	No	Inflammatory bowel disease	AMC (N = 1)	No	VAN	No	Yes
3	45/F	BGUH (Alicante)	Community	No	Chronic steroids	FQ (N = 1)	Yes	FDX	No	Yes
4	82/M	BGUH (Alicante)	Community	No	Diabetes mellitus Chronic renal failure	CEP (N = 1) FQ (N = 1)	Yes	VAN	No	Yes
5	75/F	BGUH (Alicante)	Community	No	No	CLI (N = 1) FQ (N = 1)	No	FDX	No	Yes
6	36/F	BGUH (Alicante)	Community	No	Active neoplasm	MTZ (N = 1) CEP (N = 1)	No	FDX	No	Yes
7	58/M	VBH (Orihuela)	Nosocomial	No	Chronic steroids	FQ (N = 1) AMC (N = 1)	Yes,	VAN + FDX	No	Yes

AMC: amoxycillin–clavulanic aid, BGUH Dr. Balmis General University Hospital, CEP: cephalosporin; CDI: *Clostridioides difficile* infection, CLI: clindamycin; FQ: fluroquinolones, PPI: proton pump inhibitors, MTZ: metronidazole, SCUH: San Cecilio University Hospital, VVUH: Virgen de Valme University Hospital, VBH: Vega Baja Hospital. ^a^ Recurrence defined as the reappearance of clinical symptoms compatible with CDI within 8 weeks of completing treatment, confirmed by the new detection of toxigenic *C. difficile* in stool and/or requiring treatment for CDI according to medical judgment. ^b^ Clinical cure of the episode and absence of CDI recurrence within the first 8 weeks after completing CDI treatment.

**Table 5:** Comparison of clinical and epidemiological characteristics in patients with resistant versus susceptible *Clostridioides difficile* isolates.

Variable	Resistant (N = 7)	Susceptible (N = 100)	*p* Value
Demographics			
Age in years, median (IQR)	58 (40.5–72.5)	70 (61.5–81)	0.10
Age > 79 years, n (%)	1 (14.3)	28 (28.0)	0.67
Female, n (%)	5 (71.4)	65 (65.0)	1
Clinical history and characteristics			
Charlson Comorbidity Index, median (IQR)	2 (0.5–3.5)	3 (2–4)	0.16
Previous hospitalization (<12 months), n (%)	3 (42.9)	52 (51.0)	0.71
Previous surgery (<3 months), n (%)	1 (14.3)	9 (9.0)	0.50
Previous episodes CDI, n (%)	0 (0.0)	2 (2.0)	1.0
Recent use of antibiotics (<3 months), n (%)	7 (100.0)	93 (93.0)	1.0
Use of PPI before CDI (<3 months), n (%)	3 (42.9)	67 (67.0)	0.23
Current episode of CDI			
Community-acquired, n (%)	4 (57.1)	47 (47.0)	0.80
Nosocomial, n (%)	1 (14.3)	31 (31.0)	0.65
Related to sanitary assistance, n (%)	2 (27.6)	22 (22.0)	0.65
Hospitalization, n (%)	5 (71.4)	75 (75.0)	1.00
Length of stay in days, median (IQR)	6 (6–8)	9 (6–18)	0.35
ICU admission, n (%)	0 (0.0)	2 (2.0)	1
Clinical outcomes			
Recurrence, n (%)	0 (0.0)	35 (35.0)	0.093
Fulminant/severe CDI colitis, n (%) *	1 (14.3)	26 (26.0)	0.68

CDI: *Clostridioides difficile* infection, ICU: intensive care unit, IQR: interquartile range. * Grouped severe and fulminant cases for statistical tests.

**Table 6:** Recurrence and severity of *C. difficile* infection according to minimum inhibitory concentrations (MICs), categorized into tertiles for different antibiotic therapies.

Treatment	MIC, mg/L			*p* Value ^a^	Antimicrobial Resistance Status		*p* Value ^b^
					Resistant	Susceptible	
VAN	≤0.25 (N = 36)	>0.26–0.49 (N = 48)	≥0.50 (N = 23)		(n = 3)	(n = 104)	
Recurrence, n (%)	12 (33.3)	19 (39.6)	4 (17.4)	0.175	0/3 (0)	35/104 (33.7)	0.54
Severe CDI *, n (%)	10 (27.8)	14 (29.2)	3 (13.0)	0.312	0/3 (0.0)	27/104 (26.0)	0.57
MTZ	≤0.48 (N = 36)	>0.49–<0.125 (N = 40)	≥0.125 (N = 31)		(n = 2)	(n = 105)	
Recurrence, n (%)	14 (38.9)	12 (30.0)	9 (29.0)	0.62	0/2 (0.0)	35 (33.3)	1
Severe CDI *, n (%)	9 (25)	10 (25)	8 (25.8)	0.99	0/2 (0.0)	27 (25.7)	1
FDX	≤0.06 (N = 47)	0.07–0.125 (N = 34)	>0.125 (N = 26)		(n = 1)	(n = 106)	
Recurrence, n (%)	14 (29.8)	12 (35.3)	9 (34.6)	0.04	1/01 (0)	35 (33.0)	1
Severe CDI *, n (%)	10 (21.3)	13 (38.2)	4 (15.4)	0.092	1/10 (0)	27 (25.5)	1
TGC	<0.016 (N = 37)	0.016 (N = 40)	>0.016 (N = 30)		(n = 3)	(n = 104)	
Recurrence, n (%)	27 (27)	15 (37.5)	10 (33.3)	0.614	0 (0.0)	35 (39.7)	0.54
Severe CDI *, n (%)	9 (24.3)	10 (25.0)	8 (26.7)	0.975	1 (33.39)	26 (25)	1.0
ERV	≤0.004 (N = 25)	0.005–0.008 (N = 52)	>0.008 (N = 30)				
Recurrence, n (%)	7 (28.0)	18 (34.6)	10 (33.3)	0.84	NA	NA	NA
Severe CDI *, n (%)	5 (20.0)	13 (25.0)	9 (30.0)	0.696	NA	NA	NA

ERV: eravacycline, FDX: fidaxomicin, MIC: minimum inhibitory concentration, MTZ: metronidazole, NA: not application, TGC: tigecycline, VAN: vancomycin. ^a^ *p*-values refer to the Chi-squared (2 × 3) test. ^b^ *p*-values refer to the Fisher test. * Grouped severe and fulminant cases for statistics.

**Table 7:** Participating hospitals, location, autonomous community, and number of patients included in the study.

Hospital	City/Setting	Autonomous Community	N (%)
Dr. Balmis General University Hospital	Alicante	Valencian Community	48 (44.9)
Virgen de Valme University Hospital	Seville	Andalusia	21 (19.6)
Vega Baja Hospital	Orihuela	Valencian Community	12 (11.2)
Sant Joan d’Alacant University Hospital	Sant Joan d’Alacant	Valencian Community	11 (10.3)
Virgen Macarena University Hospital	Seville	Andalucia	6 (5.6)
Marina Baixa Hospital	Villajoyosa	Valencian Community	4 (3.7)
La Fe University and Polytechnic Hospital	Valencia	Valencian Community	2 (1.9)
Reina Sofia University Hospital	Cordoba	Andalucia	2 (1.9)
San Cecilio University Hospital	Granada	Andalucia	1 (0.9)

## Data Availability

The dataset used in this study is available on request from the authors.

## Abbreviations

The following abbreviations are used in this manuscript:

Abbreviation	Definition
ERV	Eravacycline
CDI	*Clostridioides difficile* infection
FDX	Fidaxomicin
MIC	Minimum inhibitory concentrations
MTZ	Metronidazole
TGC	Tigecycline
VAN	Vancomycin
